# Self-reported medication adherence and pharmacy refill adherence among persons with ischemic stroke: a cross-sectional study

**DOI:** 10.1007/s00228-022-03284-4

**Published:** 2022-02-14

**Authors:** Helena Norberg, Maria Sjölander, Eva-Lotta Glader, Maria Gustafsson

**Affiliations:** 1 Department of Integrative Medical Biology, Umeå University, S-901 87 Umea, Sweden; 2 Department of Public Health and Clinical Medicine, Umeå University, S-901 87 Umea, Sweden

**Keywords:** Ischemic stroke, Medication adherence, Self-reported adherence, Pharmacy refill adherence, MARS-5, The Swedish Prescribed Drug Register

## Abstract

**Purpose:**

To describe and compare self-reported medication adherence assessed with the 5-item version of Medication Adherence Report Scale (MARS-5) and pharmacy refill adherence based on data from the Swedish Prescribed Drug Register (SPDR) among persons with ischemic stroke, and to investigate independent predictors associated with respective assessments.

**Methods:**

A study questionnaire was sent to persons with ischemic stroke registered in the Swedish Stroke Register between Dec 2011 and March 2012, and who lived at home 3 months after discharge. The primary outcome was dichotomized to adherent/non-adherent based on MARS-5 and SPDR and analyzed with multivariable logistic regression. Adherence according to MARS-5 was defined as score 23 or higher (out of 25). Adherence according to SPDR was defined as at least one filled statin prescription recorded in SPDR in each 6-month interval during 2 years of follow-up.

**Results:**

Of 420 participants, 367 (87%) and 329 (78%) were adherent according to MARS-5 and SPDR, respectively, and 294 (70%) participants were adherent according to both assessments. A significant association was shown between medication adherence according to the two assessments (*p* = 0.020). Independent predictors associated with medication adherence according to MARS-5 were female sex, while factors associated with SPDR were male sex and being younger.

**Conclusions:**

The majority of participants were classified as adherent, 87% according to MARS-5 and 78% based on data from SPDR. However, only 70% were adherent according to both MARS-5 and SPDR, and different predictors were associated with the different measurements, suggesting that these assessments are measuring different aspects of adherence.

**Supplementary information:**

The online version contains supplementary material available at 10.1007/s00228-022-03284-4.

## Introduction

Good adherence to medicines has a positive impact on health outcomes [1]. Unfortunately, poor medication adherence continues to be a major health challenge worldwide [2].

Prescription of statins is an established preventative treatment after ischemic stroke used to reduce recurrent cardiovascular events and mortality [3–6]. Previous studies have shown that good adherence to statin therapy is associated with reduced risk of recurrent stroke, myocardial infarction, and all-cause death [7–10]. Satisfactory adherence to statins can also improve functional outcomes in persons with ischemic stroke [11]. However, adherence to long-term treatment of chronic disease decreases with time after diagnosis [12–14], and it has been shown that up to a third of statin users are non-adherent 6 months after their stroke event [15], even though improvement of statin adherence could reduce the risk of stroke [16]. To improve medication adherence, it is important to understand that adherence to any drug therapy regimen is multifaceted and requires both motivation and ability. Non-adherence could be either unintentional (forgetting to take medication) or intentional (missing/altering doses to suit one’s needs), and these aspects of adherence have to be managed differently [2, 17].

There are several methods available for estimating adherence to drug therapy. To assess self-reported medication adherence, the 5-item version of the Medication Adherence Report Scale (MARS-5) can be applied. MARS-5 has been validated in different populations and settings, where medication refill adherence or certain surrogate markers have been used as reference [18–22]. To assess pharmacy refill adherence, it is common to analyze the drug supply, e.g., by estimating the proportion of days covered with medication or prescription refills within specified intervals. The Swedish Prescribed Drug Register (SPDR) provides data on all dispensed medicines in Swedish pharmacies and can be used to assess pharmacy refill adherence in Sweden. However, to the best of our knowledge, the relationship between self-reported adherence assessed with MARS-5 and pharmacy refill adherence assessed with SPDR has not been rigorously studied, nor has the question been asked which independent factors are related to these different methods for estimating medication adherence.

The aim of this contribution is to describe and compare self-reported medication adherence assessed with MARS-5 and pharmacy refill adherence based on data from SPDR among persons with ischemic stroke, and to investigate independent predictors associated with the two methods of assessment.

## Methods

### Design and study participants

A cross-sectional study was performed with questionnaire data on self-reported medication adherence assessed with MARS-5, clinical data from the Swedish stroke register (Riksstroke), and pharmacy refill data based on data from SPDR. The MARS-5 questionnaire and the stroke register follow-up questionnaire were sent to the study participants 3 months after their stroke events. Pharmacy refill data was limited to prescription refills of statins for up to 2 years after the participants were discharged from the hospital.

The study participants (previously described in [23]) were individuals with stroke who were registered in Riksstroke between December 2011 and March 2012. Riksstroke had an estimated coverage of 90.5% of all stroke events in 2011 and includes all 74 hospitals that treat acute stroke in Sweden. In this study, 25 out of the 74 hospitals volunteered to participate, and these institutions represented both rural and urban areas.

Inclusion criteria were as follows: persons who (i) had an ischemic stroke, (ii) were living at home 3 months after their stroke event, (iii) had a fully completed MARS-5 questionnaire, (iv) had filled at least one statin prescription within the first 6 months after discharge from the hospital, and (v) were alive at 2-year follow-up.

### Self-reported medication adherence

Self-reported medication adherence was assessed using MARS-5 [21, 24]. The MARS-5 questionnaire consists of five statements, each describing a common non-adherent behavior. The respondents rate how often they behave as described by the various statements (*I forget to take my medicines, I alter the dose of my medicines, I stop taking my medicines for a while, I decide to skip a dose, I take less than instructed*) on a 5-point Likert scale (1 = *always*, 5 = *never*). The first question focuses on unintentional non-adherence, while the other four questions reflect intentional non-adherence. MARS-5 total scores may range from 5 to 25, with higher scores indicating better medication adherence. The outcome variable was dichotomized in logistic regression analyses, according to the total score on MARS-5, where adherence was defined as scores 23–25 and non-adherence as scores 5–22.

### Prescription refill adherence

A more objective assessment of medication adherence were conducted based on retrospective pharmacy dispensation data recorded in SPDR [25]. The register was started in 2005 and provides information on all prescribed medicines dispensed at all Swedish pharmacies. The register is complete for all residents in Sweden and contains data with unique patient identifiers for all dispensed prescriptions. Information about prescriptions dispensed is transferred once per month to the National Board of Health and Welfare, which is responsible for SPDR. All drugs are classified according to the Anatomical Therapeutic Chemical (ATC) classification system. The register contains for example data on age, sex, and the history of medications prescribed and dispensed. However, information about how many days were supplied of prescribed medications is not available in SPDR.

Statins were chosen as follow-up medication because they are one of the guideline-recommended preventive drugs for persons with ischemic stroke [6]. Statins are prescribed to be taken once per day, which makes the estimates of adherence according to pharmacy refills in SPDR more reliable. The participants’ refills of statin prescriptions were followed in 6-month intervals for a maximum of 2 years after discharge from the hospital. The 6-month intervals were considered appropriate because the Swedish state subsidizes drugs if each refill corresponds to a maximum estimated 3 months’ supply, and a further grace period of 3 months were allowed in this study.

Pharmacy refill adherence to statins based on data from SPDR was used as a dichotomized outcome variable in logistic regression analyses. Participants were defined as adherent if they had at least one filled statin prescription recorded in SPDR within each 6-month interval after discharge from hospital during 2-year follow-up. Participants were defined as non-adherent if they did not refill a statin prescription in any of the remaining 3 follow-up intervals after the first 6-month period.

### Statistical analysis

Study population characteristics are presented as mean and standard deviation (SD) or frequencies and percentages. Characteristics were compared between subgroups using Student’s *t*-test for continuous variables and chi-squared test for categorical variables. The association between adherence according to MARS-5 and SPDR was examined using chi-squared test. Multivariable logistic regression models were constructed to explore independent predictors of medication adherence associated with the dependent variable adherent/non-adherent according to MARS-5 and SPDR respectively. The following independent variables were hypothesized to possibly have an impact on medication adherence and were therefore included in both regression models: age, sex, history of stroke, low level of consciousness at admission, living alone, self-reported dependence on relatives for help and support, and self-reported difficulties with memory. Age was the only continuous variable, and all other independent variables were categorical. The variable dependent on help/support from relatives were analyzed as dichotomous (yes/no), where yes included both alternatives yes (partially) and yes (completely). *P* values <0.05 were considered statistically significant. Data were analyzed in IBM SPSS Statistics V.25.0.

## Results

The study questionnaires were sent to a total of 797 people who were living at home 3 months after their stroke. Out of these 797, 594 individuals returned the questionnaires. Excluded from these 594 individuals were people who were not alive at the end of the 2-year follow-up (*n* = 34), people with stroke other than ischemic stroke (*n* = 49), people with not fully completed MARS-5 questionnaire (*n* = 49), people with no prescription of statins after discharge (*n* = 109), and people who had not filled at least one statin prescription within the first 6 months after hospital discharge (*n* = 13). After applying all inclusion criteria, 420 individuals were eligible and were included in the study.

Study population characteristics are shown in Table 1. An additional analysis showed no differences in characteristics between the original invited group (*n* = 797) versus study group (*n* = 420), or between the group that returned the questionnaires (*n* = 594) versus study group (*n* = 420) (Supplementary Table S1).

**Table 1:** Study population characteristics

**Characteristics**	**Study population (*n* = 420)**
Age (years), mean ± SD	70 ± 11
Age (years), *n* (%)	
Age < 75	262 (62)
Age ≥ 75	158 (38)
Sex, *n* (%)	
Men	253 (60)
Women	167 (40)
Low level of consciousness at admission, *n* (%)	
No	406 (97)
Yes (drowsy or unconscious)	13 (3)
History of stroke, *n* (%)	
No	355 (85)
Yes	64 (15)
*3 months follow-up (self-reported data from questionnaire)*	
Living alone, *n* (%)	
No	291 (69)
Yes	124 (30)
Dependence on relatives for help/support, *n* (%)	
No	229 (55)
Yes (partially)	152 (36)
Yes (completely)	24 (6)
Difficulties with memory, *n* (%)	
Never or almost never	170 (41)
Sometimes	190 (45)
Often or constantly	51 (12)

Of the 420 participants, 367 (87%) were categorized by the MARS-5 questionnaire as adherent and 53 (13%) as non-adherent. The mean MARS-5 score was 23.9 with a range 8–25 and was positively skewed, with half of the respondents (51%) scoring 25. An overview of the MARS-5 results is shown in Table 2.

**Table 2:** Overview of MARS-5 scores and its individual items

**MARS questions**	**Mean ± SD**	**Always = 1,** ***n* (%)**	**Often = 2,** ***n* (%)**	**Sometimes = 3,** ***n* (%)**	**Rarely = 4,** ***n* (%)**	**Never = 5,** ***n* (%)**
Item 1: “I forget to take my medicines”	4.49 ± 0.72	1 (0.2)	5 (1.2)	34 (8.1)	129 (30.7)	251 (59.8)
Item 2: “I alter the dose of my medicines”	4.82 ± 0.55	3 (0.7)	0 (0)	13 (3.1)	38 (9.0)	366 (87.1)
Item 3: “I stop taking my medicines for a while”	4.89 ± 0.42	1 (0.2)	1 (0.2)	7 (1.7)	27 (6.4)	384 (91.4)
Item 4: “I decide to skip a dose”	4.90 ± 0.38	1 (0.2)	0 (0)	6 (1.4)	26 (6.2)	387 (92.1)
Item 5: “I take less than instructed”	4.84 ± 0.53	2 (0.5)	2 (0.5)	12 (2.9)	28 (6.7)	376 (89.5)

*MARS* Medication Adherence Report Scale, *SD* Standard Deviation

**Fig. 1:**
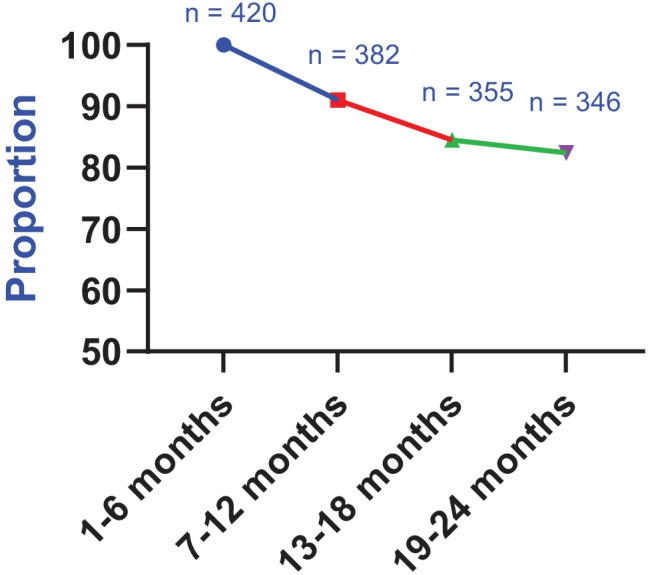
The proportion of individuals who had a registered statin prescription refill according to the Swedish Prescribed Drug Register (SPDR) in each separate 6-month interval at the 2-year follow-up (not cumulative)

According to the SPDR investigation, 329 (78%) of the 420 participants had purchased at least one statin prescription in each 6-month interval at the 2-year follow-up and were therefore classified as adherent, while 91 (22%) individuals had no record of refilling statins in one or more of the follow-up intervals and were therefore classified as non-adherent. The groups were comparable in all characteristics except age and sex, where the non-adherent group (*n* = 91) were significantly older (73 ± 11 vs 69 ± 11 years, *p* = 0.006) and had a higher proportion of women (52% vs 36.5%, *p* = 0.009) compared with the adherent group (*n* = 329) (data not shown). Over time, the number of individuals who filled a statin prescription in each 6-month interval declined (Fig. 1). In total, 364 (87%) participants refilled their statin prescriptions in three out of the four 6-month follow-up intervals, and 390 (93%) participants refilled their statin prescription in two out of the four 6-month intervals.

Table 3 presents adherence according to MARS-5 and SPDR. Overall, 294 (70%) out of 420 participants were adherent according to both MARS-5 and SPDR. Twenty percent of the respondents who were adherent according to MARS-5 were non-adherent according to SPDR, and 11% of the respondents who were adherent according to SPDR were non-adherent according to MARS-5. A significant association was shown between medication adherence according to MARS-5 and SPDR (*p* = 0.020).

**Table 3:** Self-reported adherence assessed with the Medication Adherence Report Scale (MARS-5) versus pharmacy refill adherence based on data from the Swedish Prescribed Drug Register (SPDR) (*p* = 0.020)

		**Pharmacy refill adherence (SPDR)**		
		**Yes**	**No**	**Total**
**Self-reported adherence (MARS-5)**	**Yes**	294 (80%)	73 (20%)	367 (100%)
		89%	80%	87%
	**No**	35 (66%)	18 (34%)	53 (100%)
		11%	20%	13%
	**Total**	329 (78%)	91 (22%)	420 (100%)
		100%	100%	100%

**Fig. 2:**
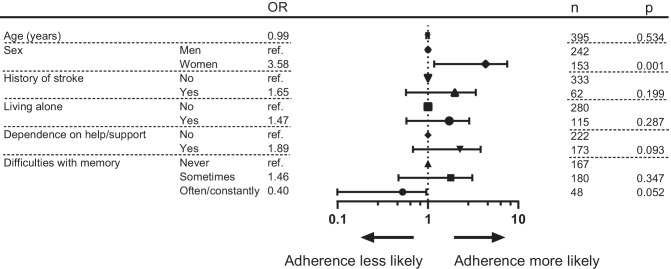
Independent predictors of medication adherence associated with the Medication Adherence Report Scale (MARS-5). Participant characteristics are listed in the two left-hand columns. The odds ratios (OR) for each variable are listed next, and these ORs are then plotted on a logarithmic scale. The two right-hand columns report the number of participants for each variable (*n*) and whether the differences between the variables was significant (*p*)

**Fig. 3:**
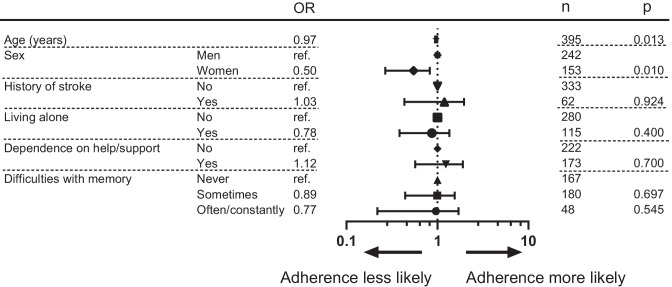
Independent predictors of medication adherence based on data from the Swedish Prescribed Drug Register (SPDR). Participant characteristics are listed in the two left-hand columns. The odds ratios (OR) for each variable are listed next, and these ORs are then plotted on a logarithmic scale. The two right-hand columns report the number of participants for each variable (*n*) and whether the differences between the variables was significant (*p*)

Multivariable logistic regression models for independent predictors of medication adherence associated with MARS-5 and SPDR are presented in Figs. 2 and 3. When medication adherence was assessed with MARS-5, women had 3.6 times higher odds of being adherent than men (OR 3.58, 95% CI 1.63–7.86). Medication adherence according to pharmacy refills from SPDR was independently associated with age and sex. For every year increase, respondents had 3% lower odds of being adherent (OR 0.97, 95% CI 0.94–0.99). Women had 0.5 times lower odds of being classified as adherent (OR 0.50, 95% CI 0.29–0.85) than men.

## Discussion

The majority of persons with ischemic stroke adhered to their prescribed statins, according to both self-reported medication adherence (MARS-5) and as assessed by prescription refills based on data from SPDR. However, only 70% of the participants were adherent both according to MARS-5 and according to SPDR, which indicates that these assessments probably represent different aspects of medication adherence. Differences between the two types of assessments were also shown in terms of independent predictors where medication adherence according to MARS-5 was associated with female sex, while medication adherence based on data from SPDR was associated with male sex and being younger.

### Adherence to prescribed statins

Our results show a slightly higher adherence compared with previous studies for persons with ischemic stroke. One reason could possibly be that the included participants were more adherent to statin therapy because the population only included those who fulfilled the questionnaire MARS-5, living at home at 3 months and were alive 2 years post stroke and filled a prescription within 6 months of discharge. However, different follow-up times and methods used to assess adherence make it difficult to directly compare these studies [26]. In Yao et al. [27] and Wawruch et al. [28], approximately 60% of participants with atherosclerotic cardiovascular disease or ischemic stroke were adherent to statins according to prescription refills after 1 year and 3 years, respectively. Chung et al. [15] assessed self-reported statin adherence with the 8-item Morisky Medication Adherence Scale in patients with acute ischemic stroke, showing that 66% were adherent after 6 months.

Studies with statins for primary and secondary prevention of coronary artery disease have shown that prescription refill adherence at the 2-year follow-ups was 25% and 40%, respectively [29]. Naderi et al. [12] conducted a meta-analysis including more than 370,000 participants with or without coronary heart disease, showing that adherence to statin therapy was 57% and 76% for primary and secondary prevention after a median treatment period of 2 years, which is similar to our results.

Regarding the choice to study statins in the present study, and not, e.g., antihypertensives or anticoagulants also used in ischemic stroke prevention, were based on previous studies showing no significant differences in adherence or persistence to treatment between statins, anticoagulants, and antihypertensives [30, 31]. Further, because the SPDR does not contain data of daily doses, the estimates of adherence from SPDR were possible to perform more precisely in statins that merely are prescribed once per day.

### Various assessments of adherence

Twenty percent of the respondents who were adherent according to self-reported adherence with MARS-5 were non-adherent according to pharmacy refills based on data from SPDR. Previous studies have shown that self-reported adherence assesses medication-*taking* behaviors, whereas pharmacy refill adherence assesses the medication-*filling* behaviors [32]. MARS-5 assesses whether individuals take their drugs after they are purchased (and can give insights into reasons for non-compliance), whereas SPDR counts the frequency of medication-filling behavior (that is, whether individuals fill their prescriptions in the first place, and then refill them over specified time intervals) [32, 33]. Even though these two assessments measure different aspects of medication adherence, it is clear that they can complement each other.

Our results are in line with previous studies that have shown that participants tend to overestimate their self-reported adherence to drug treatment or prescription filling compared to more objective assessments [33–39]. Furthermore, participants who are adherent according to self-reported assessments but non-adherent according to assessments that are more objective are, in some studies, defined as “false-positive self-reported adherers”. For instance, Tedla and Bautista [40] studied factors associated with false-positive self-reported adherence in 175 individuals who were starting antihypertensive drug therapy in primary care. In that study (and in accordance with our results), 20% of the participants were classified as false-positive self-reported adherers. That study also found that falsely over-reported adherence was more likely in participants with anxiety and low educational levels, and less likely among smokers and those who suffered from blood pressure drug side effects.

The methods currently available for determining medication adherence differ widely [41, 42]. It is common in previous studies to assess medication availability, such as the proportion of days covered during a specified time period or over a period of refill intervals. In addition to the various methods, different terminology is also used. In this study, the term “pharmacy refill adherence” were chosen because it best fit the data obtained from SPDR. When estimating pharmacy refill adherence and choosing the length of the follow-up intervals, it is important to consider how poor implementation of the treatment or the length of gaps between treatments will affect the therapeutic benefit; for shorthand, these considerations are called the “forgiveness” of the drug [43]. In contrast to, e.g., antiretroviral drugs and anticoagulants, a short, temporary lapse in treatment of statins does not affect the therapeutic effect — in other words, statins are a “forgiving” treatment. Forgiving treatments have a cumulative effect: the better the implementation and persistence with treatment, the higher the benefit. In drugs with high forgiveness, it is possible to apply a longer grace period [41] and in that way minimize the risk of overestimating the non-adherence rate. To avoid overestimating non-adherence in the present study, a 3-month grace period was chosen in addition to the 3-month pharmacy refill intervals.

### Independent predictors of medication adherence

In the multivariable logistic regression models, female sex was associated with medication adherence according to MARS-5, while male sex and younger age were associated with medication adherence based on data from SPDR. Several large studies and meta-analyses have shown that female patients are consistently more likely to be non-adherent to statin therapy and other cardiovascular long-term therapies when assessed with objective assessments [44–47]. However, in a validation study of MARS-5, female sex was associated with higher MARS-5, as well as a more objective assessment with medication possession rate [19].

The association between age and medication adherence has previously been studied. In a meta-analysis of 22 cohort studies, a U-shaped pattern of the relative risk of non-adherence was shown; the youngest (< 50 years) and oldest (≥ 70 years) had lower adherence as assessed with percentage of days covered, compared with the middle-aged (50–69 years) [46]. The study population in our study had an average age of 70 ± 11 years and considering that the number of drugs increase with age [45], polypharmacy could possibly be a predictor for non-adherence among included individuals.

The study questionnaire contained some questions in which the respondents were asked to rate their current activity level or amount of help they need, e.g., dependence on relatives for help and support and difficulties with memory. These two variables did not show association with medication adherence in either of the multivariable analyses based on data from either MARS-5 or SPDR, although there was a trend towards lower adherence in participants who reported that they had often or always difficulties with memory in MARS-5. This result can probably be explained by the low need for help from relatives among the participants; the majority of these reported that they only needed partial support. In addition, only 12% rated that they often or always had difficulties with memory.

Other studies have revealed that a combination of patient and medical factors affect adherence to statin therapy. McGinnis et al. [48] showed that the most common reasons for statin discontinuation were follow-up and/or laboratory visits with a provider within 6 months after the statin prescription was initiated; adverse events, patient refusal, worry about developing adverse effects, the patient felt the treatment was unnecessary, physician-advised discontinuation and the patient preferred to manage their condition using diet and exercise. Similarly, Chee et al. [49] found that the major barriers to statin adherence were failure to appreciate the severity of potential complications, lack of perceived benefits, adverse events, medication cost, poor physician–patient relationship, and overestimation of the effectiveness of diet control as a treatment modality.

### Limitations

The participants responded to the MARS-5 questionnaire 3 months after their stroke event, while SPDR followed up on refilled statin prescriptions during the 2 years post stroke. Because medication adherence often declined with time after diagnosis [12–14], the time aspect of the two assessments could have affected the results. The MARS-5 questionnaires presented to the participants considered their total medication list, not particular treatments or specific medications such as statins. In this study, an assumption was made that the participants were equally adherent to statins as to other long-term treatments that was measured by MARS-5. However, adherence may differ in some participants between different long-term drugs [50].

Only individuals who were alive 2 years after suffering mild ischemic stroke, who were conscious at stroke admission, and who were living at home 3 months after discharge were included. Therefore, the results cannot be extrapolated to the most frail patients, including those whose conditions deteriorated after the stroke or individuals with severe ischemic stroke. Likewise, other relevant cultural and socio-economic aspects differ between countries and the results may not be generalizable to populations outside Sweden.

## Conclusions

This study found that the majority of the participants were classified as adherent, 87% according to MARS-5 and 78% based on data from SPDR. However, only 70% of the participants were adherent according to both MARS-5 and SPDR, and different predictors were associated with the different measurements. This result suggests that these assessments are measuring different aspects of adherence.

## Supplementary information

Below is the link to the electronic supplementary material.

Supplementary file1 (DOCX 23 KB)

## Data Availability

The datasets used and/or analyzed during the current study are available from the corresponding author on reasonable request.
