# Supplementary material for: Self-reported medication adherence and pharmacy refill adherence among persons with ischemic stroke: a cross-sectional study
Source: Eur J Clin Pharmacol. 2022 Feb 14;78(5):869–77. doi: 10.1007/s00228-022-03284-4 (PMC9005421; doi:10.1007/s00228-022-03284-4)
Supplement: Supplementary file 1 — Supplementary file1 (DOCX 23 KB) [file 228_2022_3284_MOESM1_ESM.docx]

## Supplementary Information

**Table S1.** Characteristics comparison between the subgroups who were sent the study questionnaires (n=797) and the subgroup that returned the questionnaires (n=594) versus the study population (n=420) respectively.

| **Characteristics** | **n=797ƚ** | **n=594ǂ** | **n=420** | **P value** |
| --- | --- | --- | --- | --- |
| Age (years), mean ± SD | 71 ± 12 | 71 ± 12 | 70 ± 11 | 0.058ƚ, 0.228ǂ |
| Age (years), n (%) |  |  |  | 0.056ƚ, 0.153ǂ |
| Age <75 | 452 (57) | 344 (58) | 262 (62) |  |
| Age ≥75 | 354 (43) | 250 (42) | 158 (38) |  |
| Sex (n (%) |  |  |  | 0.420ƚ, 0.922ǂ |
| Men | 461 (58) | 356 (60) | 253 (60) |  |
| Women | 336 (42) | 238 (40) | 167 (40) |  |
| Low level of consciousness at admission, n (%) | |  |  | 0.330ƚ^a^, 0.432ǂ^f^ |
| No | 754 (95) | 564 (95) | 406 (97) |  |
| Yes (drowsy or unconscious) | 38 (5) | 28 (5) | 13 (3) |  |
| History of stroke, n (%) |  |  |  | 0.772ƚ^b^, 1.000ǂ^g^ |
| No | 666 (84) | 503 (85) | 355 (85) |  |
| Yes | 130 (16) | 90 (15) | 64 (15) |  |
| *3 months follow-up (self-reported data from questionnaire)* | |  |  |  |
| Living alone, n (%) |  |  |  | 0.198ƚ^c^, 0.974ǂ^h^ |
| No | 516 (65) | 412 (69) | 291 (69) |  |
| Yes | 274 (34) | 176 (30) | 124 (30) |  |
| Dependence on relatives for help/support, n (%) | |  |  | 0.089ƚ^d^, 0.250ǂ^i^ |
| No | 385 (48) | 295 (50) | 229 (55) |  |
| Yes (partially) | 310 (39) | 234 (39) | 152 (36) |  |
| Yes (completely) | 72 (9) | 48 (8) | 24 (6) |  |
| Difficulties with memory, n (%) |  |  |  | 0.869ƚ^e^, 0.834ǂ^j^ |
| Never or almost never | 311 (39) | 232 (39) | 170 (41) |  |
| Sometimes | 358 (45) | 279 (47) | 190 (45) |  |
| Often or constantly | 108 (14) | 74 (13) | 51 (12) |  |

^a^ n=1211, ^b^ n=1215, ^c^ n=1205, ^d^ n=1172, ^e^ n=1188, ^f^ n=1011, ^g^ n=1012, ^h^ n=1003, ^i^ n=982, ^j^ n=996
